# Mapping the Healthiness of Campus Food Environments in Nigeria: In-Store Retail and Outdoor Advertising Assessments at Selected Universities—The NigeFE Study

**DOI:** 10.3390/ijerph23081052

**Published:** 2026-08-13

**Authors:** Gideon Onyedikachi Iheme, Ogechi Chinyere Nzeagwu, Anuoluwapo Funmilayo Taiwo, Uju Maryanne Onuorah, Amarachi Precious Nwonu, Temitope Bodunde Ayegbusi, Gift Isaiah, Chioma Onuoha Igboamagh, Flourish Chizurum Stephen, Nnenna Hope Ugwu, Chinyere Ikejiofor

**Affiliations:** 1 Department of Food Studies, Nutrition and Dietetics, Uppsala University, SE-751 22 Uppsala, Sweden; 2 Department of Human Nutrition and Dietetics, Michael Okpara University of Agriculture, Umudike 440109, Nigeria; 3 Department of Human Nutrition and Dietetics, University of Ibadan, Ibadan 200005, Nigeria; 4 Department of Nutrition and Dietetics, University of Nigeria, Nsukka 410001, Nigeria; 5 Department of Pharmacy and Health and Nutrition Sciences, University of Calabria, 87036 Rende, Italy; 6 Department of Nutrition and Dietetics, Alex Ekwueme Federal University Teaching Hospital, Abakaliki 480282, Nigeria; 7 Department of Nutrition and Dietetics, School of Technology, Yaba College of Technology, Yaba 101212, Nigeria; 8 Nutrition Unit, National Agency for Food and Drug Administration and Control, Isolo, Lagos 102214, Nigeria

**Keywords:** campus/university, food environment, food retail, advertisement, prepackaged foods, NigeFE

## Abstract

**Highlights:**

**Public health relevance—how does this work relate to a public health issue?**

- In a predominantly youth population, the campus food environment remains a key contributor to shaping dietary patterns and the overall risk of NCDs among young adults.
- Evidence on the healthiness of campus food environments (food ads and retail) in low- and middle-income countries remains limited in this region, as the few emerging insights have been conducted largely in lower education settings or focused on specific indicators.

**Public health significance—why is this work of significance to public health?**

- This study provides one of the first comprehensive assessments of campus food environments in selected Nigerian institutions by integrating outdoor food advertising, retail food availability and affordability alongside geospatial mapping.
- The findings reveal a predominance of unhealthy food advertising, limited availability and affordability of healthy foods, and significant differences in food environments across universities.

**Public health implications—what are the key implications or messages for practitioners, policy makers and/or researchers in public health?**

- There is a need to implement and enforce strong regulations that make healthy foods available at subsidised rates while restricting the marketing and sales of less healthy alternatives.
- The study provides evidence to support campus food environment interventions and highlights the value of geospatial mapping for informing nutrition policies and future food environment research.

**Abstract:**

The rising burden of obesity and non-communicable diseases among young people has prompted global recommendations for healthier food environments, but evidence is limited across African universities. This study aims to assess the healthiness and spatial distribution of university food environments using in-store retail audits and outdoor food advertising assessments. Outdoor food advertisements were audited in two universities (excluding the University of Ibadan), while retail stores were assessed across three participating institutions within a 300–500 m radius of each campus. Data were collected using the INFORMAS protocol and the NEMS-S-MED in-store audit tool, respectively, alongside spatial analyses. A total of 76 food advertisements and 145 food retail stores were identified. Less than one-tenth (7.5%) of advertised products were healthy. Healthier foods in retail stores were predominantly characterised by moderately low (37.2%) and medium (31.7%) availability, while affordability was low (73.1%), resulting in over half being classified as having a low (51.7%) degree of healthiness. These differed significantly across institutions (*p* < 0.05), with the University of Nigeria, Nsukka (UNN) having a more diverse, spatially dispersed and healthier food environment. There is a need to restrict unhealthy food advertising and encourage vendors’ procurement and the sale of healthier foods.

## 1. Introduction

The university environment represents a key setting for the expression of independence, as many young people leave their parental homes for the first time and enter contexts where they exercise substantial autonomy over multiple aspects of their well-being, including food choices and dietary practices [1,2]. The mandatory minimum university entry age of 16 years in Nigeria [3], and the median graduation age of 22–23 years reported in Europe [4], underscore a transitional developmental period spanning late adolescence and early adulthood, during which self-adopted behaviours are formed and are likely to persist across the life course [5]. Navigating this complex life phase is further complicated by the demands of academic schedules combined with extracurricular commitments and, in some cases, income-generating activities such as part-time employment or business ventures [6,7], fostering a natural preference for convenient approaches and coping mechanisms to daily activities, which may have implications for dietary and lifestyle habits [8,9].

Due to globalisation, the vacuum created by work and academic scheduling appears to be filled by the growing penetration of pre-packaged foods, which offer convenience in exchange for the time and effort required in traditional food preparation [10]. Evidence suggests that university retail food environments are increasingly dominated by unhealthy food options, characterised by high levels of fat, sugar, and sodium, as well as low micronutrient density [11,12]. Because campus food retail services are often outsourced to commercial, profit-driven entities [13,14], unhealthy food products are not only made widely available for sale within retail outlets but are also heavily promoted and marketed in African schools and campuses [15,16,17].

These poor food environments are associated with unhealthy dietary choices, which have been linked to obesity and non-communicable diseases [18,19], adverse academic performance [20], and a negative environmental impact [21]. Thus, effective strategies for stimulating the sustained adoption of healthy practices are rooted in structural changes that restrict physical and economic access to unhealthy foods and their associated promotional strategies [22,23]. However, robust evidence demonstrating the extent to which the evolving African food environment is reshaping public institutions is required to underscore the urgency of action [24,25]. While substantial evidence exists on university food environments in high-income settings, there remains a marked paucity of data from Africa’s contexts, where food environment research has been limited to generic retail stores [26,27,28], and outdoor food advertising studies in schools have largely focused on lower levels of education [15,16]. Therefore, this study aims to examine the healthiness of university food environments through a comprehensive in-store retail audit, outdoor advertising assessment and spatial analysis across selected Nigerian universities.

## 2. Methods

### 2.1. Study Design and Settings

This study employed a cross-sectional audit of the campus food environments in three Nigerian universities: the University of Nigeria, Nsukka (UNN), Michael Okpara University of Agriculture, Umudike (MOUAU), and the University of Ibadan (UI).

These institutions were purposively selected to include universities located in different geographical and campus contexts. Specifically, the selected universities represent rural/semi-urban (MOUAU and UI) and urban (UI) settings across two geopolitical zones in southern Nigeria, enabling comparisons of campus food environments across different contextual settings.

### 2.2. Data Collection and Coding

Data was collected between April 2024 and May 2024 using structured and validated audit tools for outdoor food advertisement and retail in-store food environment developed by INFORMAS (International Network for Food and Obesity/Non-Communicable Diseases Research, Monitoring and Action Support) [29] and the Nutrition Environment Measures Survey in Stores for Mediterranean Contexts (NEMS-S-MED) [30]. The University of Ibadan was not included in the outdoor food advertisement component of the data collection because of operational constraints during the survey period.

Open Data Kit, an open-source software for data collection and management in resource-constrained settings, was utilised to digitally collect data on-campus food environment [31,32]. This web and app-based platform, integrated with a Global Positioning System (GPS)- enabled device, aided the collection of all relevant geographic coordinates (latitude and longitude, altitude, and positional accuracy) for both outdoor food advertisements and retail food stores within a 300–500 m radius of each campus. The 300–500 m radius was selected to capture areas within walking distance of the campuses, including nearby off-campus student residences, and was consistent with the recommended scope of the INFORMAS outdoor food advertisement protocol [29].

All outdoor food advertisements were audited, except those located inside retail stores. For retail food outlets, all stores stocking a broad range of food products within the study area were included, whereas most open-air vendors and specialty stores were excluded because they stocked only a limited range of food groups.

Data collection was conducted by a team comprising two lead institutional researchers and three to five trained research assistants at each participating institution. All data collectors had expertise in nutrition and food environment research and received standardised training on the use of the ODK platform, the INFORMAS Food Promotion protocol, the NEMS-S-MED retail audit tool, and the associated spatial data collection procedures. To ensure consistency across institutions, the study team used standardised data collection protocols and conducted supervised field practice before the commencement of data collection.

Outdoor food advertisements comprise all forms of advertisement strategically situated in outdoor areas, within the campus or its surrounding area (about 300–500 m radius around the schools). The elicited information followed the INFORMAS protocol for recording outdoor food advertisements [29]. These include advertisement size (small (0.21 m  ×  0.30 cm–1.3 m  ×  1.9 m)), medium (>1.3 m  ×  1.9 m–2.0 m  ×  2.4 m), large (≥above 2 m  ×  2.5 m), advertisement settings (food shop, roads, building, bus park etc.), the type and position of the advertisement (billboard, poster, free-standing, painted, digital/LED etc.), promotional characters (cartoon/company-owned characters, amateur sportsperson, famous sportsperson/team, celebrity/famous (non-sports) etc.) and premium offers (game and app downloads, contests, pay two–take three or others, 20% extra or other, price discounts and loyalty programmes). As an advert could contain multiple products, the following product specific characteristics were obtained: number of food products per ad, product type and brand name, food product healthiness category (healthy/core, unhealthy/non-core and miscellaneous), food product categories (confectionary, bread and bakery products, cereal and cereal products, dairy, edible oils and oil emulsions, fruit and vegetables etc.). The food healthiness was classified as healthy (core) or unhealthy (non-core) based on a range of predefined criteria primarily related to their composition of nutrients of concern, such as sugar, salt, sodium, cholesterol, fats, saturated fats, and dietary fibre at critical levels in specific food items. Foods not listed in healthy or healthy groups were classified as miscellaneous. All advert and product characteristics have been described in detail elsewhere [29].

The NEMS-S-MED is an observational assessment of the nutrition environment in retail food stores (NEMS-S) to identify the availability of healthier foods and their varieties, as well as the affordability of these healthier options compared with less healthy alternatives. The foods contained in the NEMS-S-MED survey instrument were drawn from 12 main food groups: (i) vegetables, (ii) fresh fruits, (iii) nuts, (iv) non-alcoholic beverages, (v) bread, cereals, and baked goods, (vi) milk and dairy products, (vii) eggs, (viii) oil and butter, (ix) rice, (x) legumes, (xi) meat and meat products, and (xii) fish and fish products.

A few modifications were made to these survey instruments to align with local context. Local foods that did not fit the predefined food categories of the INFORMAS Food Promotion and NEMS-S-MED protocols were assigned to the most closely matching food group. For example, tuber-based products were classified under the closest comparable category (i.e., cereals and cereal products) in the food advertisement audit. However, these products were not included as separate categories in the retail food store product list to maintain consistency with the original protocol and facilitate comparability across different settings and regions. Furthermore, given the diversity of retail food stores across the participating institutions, response options such as “None”, or “Not applicable” were included to indicate the absence of specific food groups or assessment components within individual stores. Consequently, the fruit and vegetable variety category was modified from “<5 varieties” to “1–5 varieties” to accommodate stores with no fruit, vegetable or fish offerings.

Individual measures of the retail food assessment were scored, with higher scores indicating greater availability or affordability of healthier foods. For availability, fruit varieties, fresh vegetables, frozen vegetables, fresh fish, and frozen fish were each assigned a maximum score of 4 points based on the number of varieties available in each retail store: >10 varieties = 4 points, 6–10 varieties = 3 points, and 1–5 varieties = 2 points. Five food groups—nuts, non-alcoholic beverages, eggs, oils and butter, and legumes—each comprised a single healthier comparison item and were assigned 1 point when that item (raw nuts, 100% fruit juice, eggs, extra-virgin olive oil, or legumes) was available. The remaining food groups comprised two or three healthier comparison items, with each item assigned either 1 or 2 points. These included bread, cereals and baked goods (100% whole-grain bread = 1 point; low-sugar cereals = 1 point), milk and dairy products (any milk = 1 point; skimmed milk = 1 point; cheese = 1 point), rice (whole-grain rice = 2 points; white rice = 1 point), and meat and meat products (red meat = 1 point; poultry = 2 points).

The affordability assessment focused on the relative affordability of healthier food options compared with their less healthy counterparts. Fruits, vegetables, fish, nuts, and legumes in their raw, fresh, or frozen forms were excluded because no comparable less healthy alternatives were available. A maximum score of 2 points was assigned for each affordability comparison. Single-comparison food groups included bread, cereals and baked goods (low-sugar cereals ≤ regular cereals), rice (whole-grain rice ≤ white rice), and meat and meat products (poultry ≤ red meat), each scoring 2 points when the healthier option was equally or more affordable. Food groups with two affordability comparisons included non-alcoholic beverages (diet soda ≤ regular soda = 2 points; 100% fruit juice ≤ juice drinks = 2 points) and milk and dairy products (skimmed or low-fat milk < whole milk = 2 points; skimmed or low-fat milk = whole milk = 1 point).

These individual-level items culminated in a composite availability and affordability score of 37 and 12, respectively. Food availability was categorised into: low availability (score 1–7), moderately low availability (8–14), medium availability (15–22), moderately high availability (23–30), and high availability (31–37). Similarly, the affordability of healthy food relative to less healthy options was graded as: Low Price (0–3), Moderate Price (4–7), High Price (8–12). A merger of the food availability and food affordability scores yielded an overall healthiness score ranging from 0 to 49, graded as Low (0–16), Moderate (17–33), and High (34–49). All scoring approaches followed the procedure stipulated in the NEMS-S-MED protocol [30].

For spatial mapping, points were classified by the predominant healthiness of advertised food products (healthy vs. unhealthy), and marker sizes were scaled to reflect the number of food products displayed per advert. On the other hand, retail stores were categorised by healthiness level (low vs. moderate–high).

### 2.3. Data Analysis

Descriptive statistics (frequencies, percentages, mean and standard deviation) were used to summarise the distribution of food advertisements, availability and affordability of healthy foods, and overall healthiness scores.

A comparative analysis (Chi-square tests and Kruskal–Wallis) was conducted to identify significant differences in various food environment characteristics (*p* < 0.05) across the selected campuses (*p* < 0.05).

Separate campus-level maps were generated for each university and arranged side-by-side within a single figure to facilitate visual comparison across institutions. Shared legends were applied to ensure consistency in classification and interpretation. Final maps were exported as high-resolution images for reporting and dissemination.

Statistical analyses were conducted using IBM SPSS (Version 27). Spatial analysis and map visualisations were conducted using Python (Version 3.12) frameworks, GeoPandas (Version 1.1.4) for Spatial data handling and Matplotlib (Version 3.11.0) for plotting.

## 3. Results

A comprehensive audit was conducted, covering 145 food retail stores across three university campuses and 76 food and beverage advertisements from two universities. The University of Nigeria, Nsukka (UNN) accounted for the majority of the advertisements (79.0%), while the Michael Okpara University of Agriculture, Umudike (MOUAU) contributed to the remaining (21.0%). In terms of in-store assessments, UNN had the highest proportion of retail stores (45.0%), followed by MOUAU (28.3%) and the University of Ibadan (UI) (26.7%).

### 3.1. Advertisement Type and Settings

Results from Table 1 revealed the type and settings of food advertisements across the two institutions. Most (67.1%) of the advertisements were small in size, predominantly posters or banners (64.5%), and were found within the vicinity of food shops (77.6%). This varied significantly (*p* < 0.05) across the studied institutions, as reports showed that while majority (81.3–100%) of the adverts in MOUAU were small-sized posters/banners, and found within food shop, UNN also contained a variety of other alternatives for advert size (medium size—36.7%), type (free-standing—30.0%) and settings (buildings—13.3%).

A good number of the adverts incorporated promotional features, marked by the presence of promotional characters (39.5%) and premium offers (38.2%). No persuasive advertising techniques were identified at MOUAU.

### 3.2. The Healthiness of Food Advertised by the School

Information on the healthiness of pre-packaged food products advertised across various food categories and institutions is summarised in Table 2. The results revealed a mean (SD) of 1.63 ±1.48 products were identified per advertisement, with a range of 1–6 products for MOUAU and 1–8 products in UI. This culminated in an overall count of 120 food products displayed across the 76 adverts. The results showed that the majority (85.8%) of the advertised pre-packaged foods were classified as unhealthy. In comparison, less than a tenth (7.5%) were classified as healthy, and the remaining 6.7% were classified as miscellaneous. Across institutions, MOUAU (12.5%) had more healthy products than UNN (6.3%), while UNN (85.4%) had a slightly lower proportion of non-healthy foods. No products in MOUAU (0.0%) were classified as miscellaneous.

**Figure 1:**
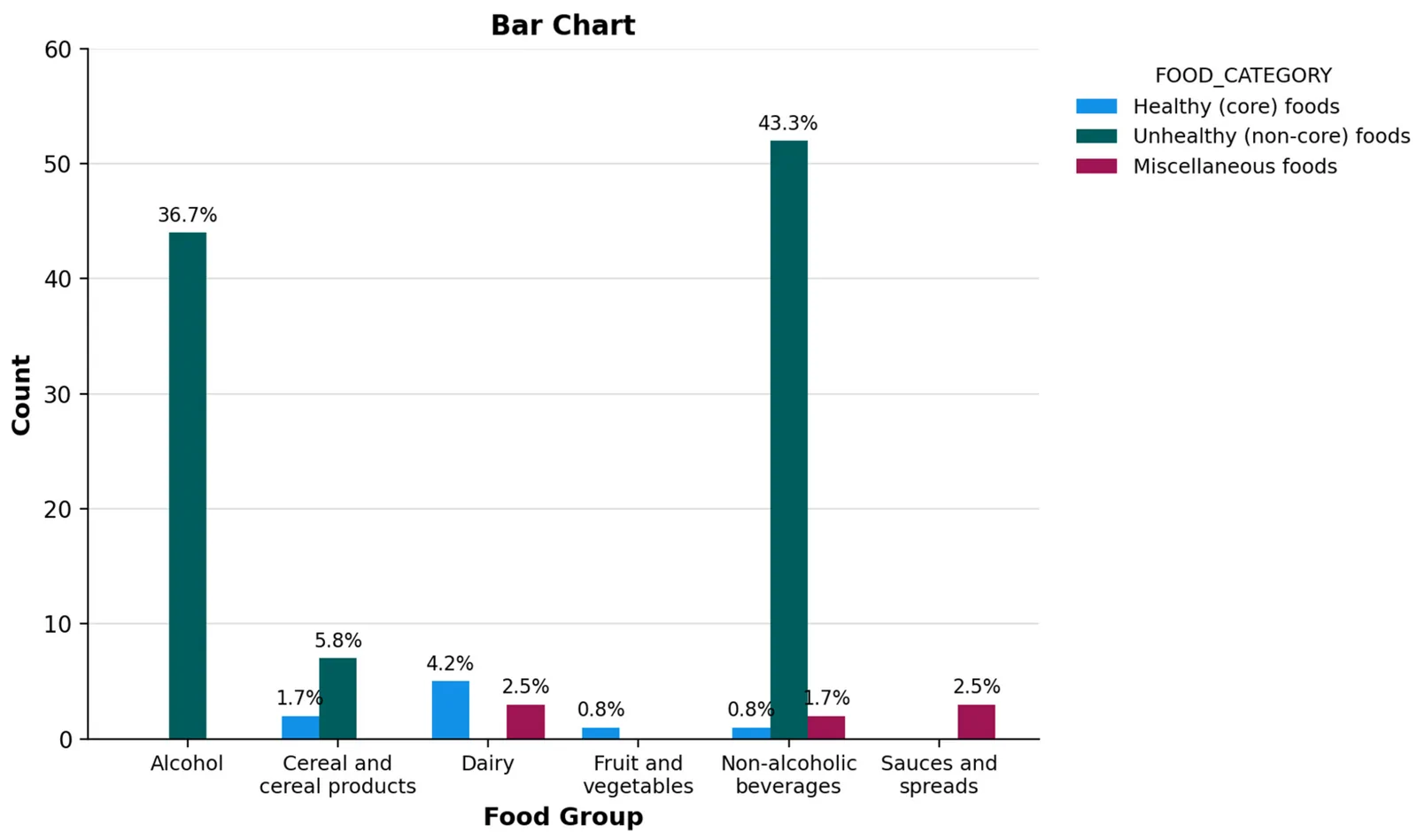
Distribution of the healthiness of advertised pre-packaged foods across food categories.

The advertised products were predominantly unhealthy beverages, including non-alcoholic (n = 52; 43.3%) and alcoholic (n = 44; 36.7%) drinks. On the other hand, dairy products (n = 8; 6.7%) and cereal-based items (n = 9; 7.5%) were considerably less represented (Figure 1).

**Figure 2:**
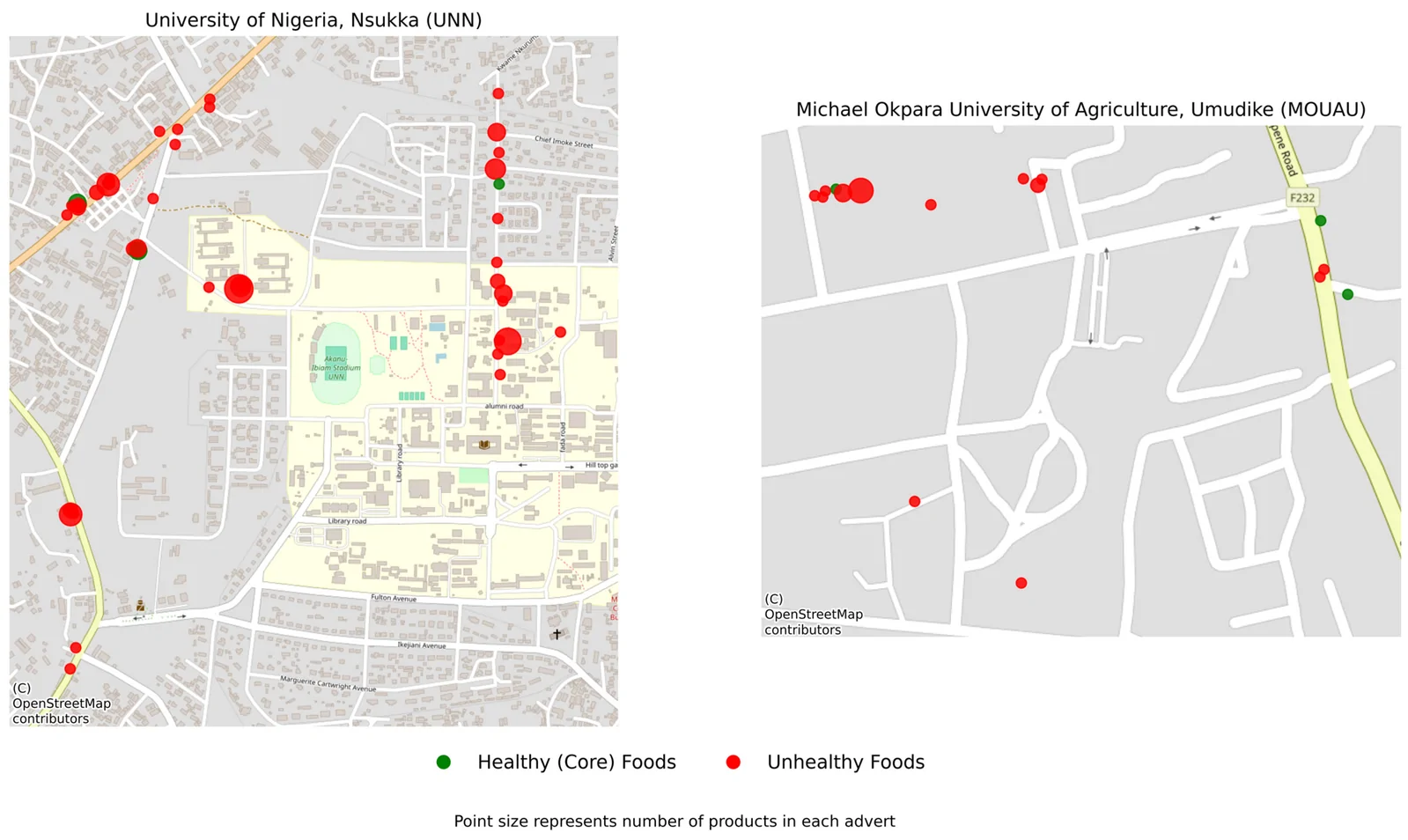
Spatial distribution of food advertisements’ healthiness across selected Nigerian university campuses. Basemap: OpenStreetMap contributors (Open Database License, ODbL). The maps were generated by the authors using Python (Version 3.12) under the applicable licence conditions.

These predominantly unhealthy food advertisements were spatially clustered in both institutions, with a marginally broader spatial distribution and multiple products per advert observed at UNN relative to MOUAU (Figure 2).

### 3.3. Availability of Healthy Foods at the Campus Retail Stores

Table 3 shows the availability of healthy food items within the university environments.

The majority of the stores reported having limited (1–5) varieties of fresh fruits (56.6%), fresh vegetables (57.2%), frozen vegetables (75.2%), fresh fish (75.2%), and frozen fish (74.5%) available. Approximately one-tenth of the stores stocked 100% juice, 100% whole-grain bread, and skim milk yoghurts. Additionally, red meat (28.3%), chicken and non-egg poultry (29.0%), cheese (17.2%), canned tuna (22.8%), and whole rice (33.1%) were in short supply. However, the most commonly available items include milk (77.2%) and eggs (80.7%), while a good number of stores stocked extra-virgin oil (44.4%) and legume products (37.9%).

There was also reportedly a significant difference across institutions (*p* < 0.001), with UNN having a higher proportion of these healthier food options than the other institutions, except for meat products, skimmed yoghurt, frozen fish varieties, and 100% fruit juice.

### 3.4. Affordability of Healthy Foods in Retail Stores

Table 4 presents information on the availability of healthy food in campus retail stores.

Although 100% juices were unavailable for comparison, a higher proportion of regular carbonated and fruit drinks (57.2%) were found to be cheaper than diet soda. In contrast, about a third (33.1%) of retail stores offered diet soda at a lower price than regular soft drinks.

The majority (70.3%) of the regular bread and cereals were generally more affordable than low-sugar alternatives. Similarly, most (64.1%) of the whole milk was found to be cheaper than skimmed or low-fat milk. About half of stores offered white rice (50.3%) and red meat (56.6%) at lower prices than healthier whole-grain rice and poultry meat, respectively. Furthermore, approximately one in ten retail stores did not stock alcoholic beverages (9.7%), bread, cereals and baked products (11.7%), or milk and dairy products (9.7%), while more than one-fifth did not stock rice (23.4%) or meat and meat products (29.0%).

Compared to other institutions, healthy foods such as poultry meat, low-sugar (whole) bread, cereals, and diet soda were significantly more affordable in UNN. Additionally, UI and MOUAU offered cheaper, healthier alternatives for dairy products and rice, respectively (*p* < 0.001).

### 3.5. Overall Healthy Food Availability, Affordability and Healthiness Scores

The summary data on the overall categories of the extent of availability, affordability and healthiness of the campus food environment is presented in Table 5.

Results show that about a third each of the selected stores fell within the moderate low availability (37.2%) and medium availability (31.7%) categories, while only a small proportion had extreme levels of high (16.6%) and low (14.5%) availability of healthy foods.

The majority (73.1%) of the healthy food options were categorised as low in affordability, with 22.1% and 4.8% classified as moderate and high affordability, respectively.

Overall, the healthiness of in-store food environments was predominantly low (51.7%) or moderate (44.8%), with very few stores (3.4%) attaining high healthiness scores.

These were significantly different across institutions (*p* < 0.001), with UNN attaining comparatively higher food availability, food affordability, and food healthiness scores.

**Figure 3:**
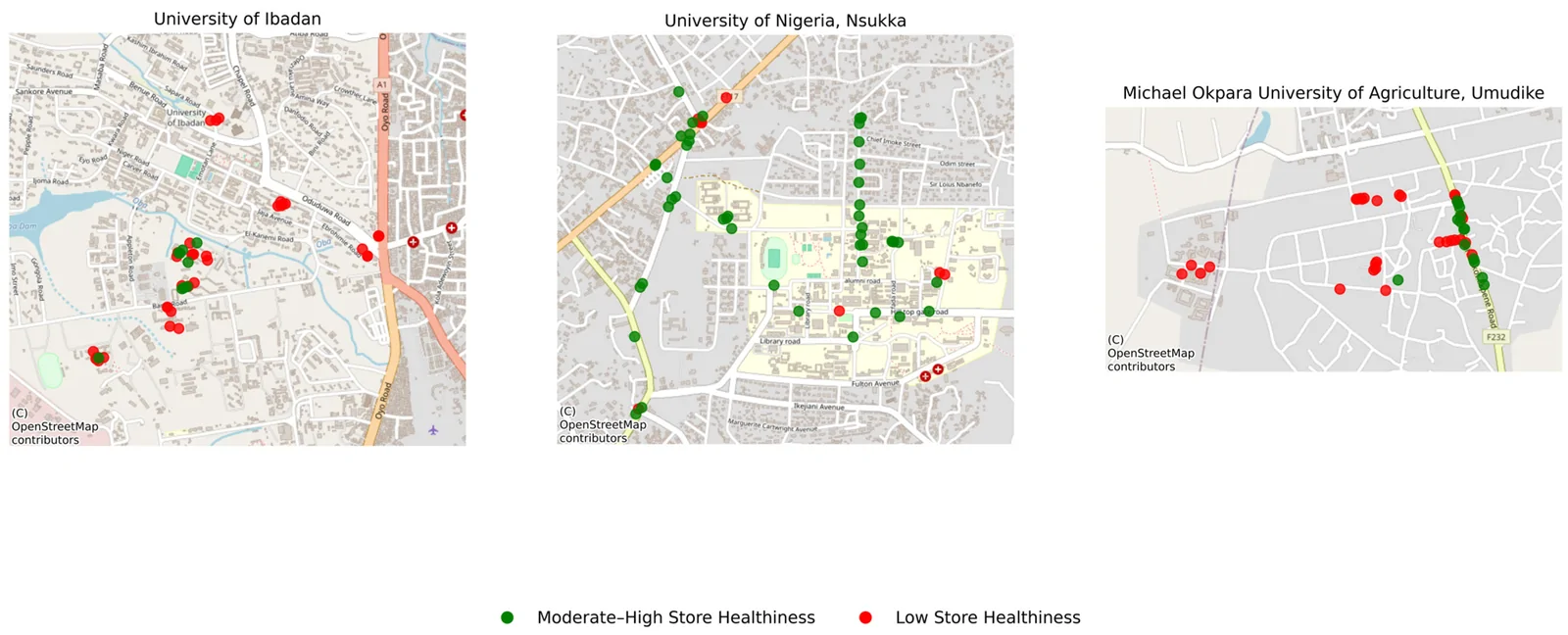
Spatial distribution of store healthiness levels across selected Nigerian university campuses. Basemap: OpenStreetMap contributors (Open Database License, ODbL). The maps were generated by the authors using Python (Version 3.12) under the applicable licence conditions.

The spatial analysis disaggregated by institution further visually reinforced that retail stores at the University of Nigeria, Nsukka (UNN) demonstrated substantially higher availability and affordability of healthy foods, alongside a more widespread spatial distribution of moderately healthy outlets across the campus. On the other hand, the other institutions exhibited comparatively lower availability and affordability of healthy foods, with retail outlets more spatially clustered and with limited coverage. In addition, UNN and UI predominantly had their retail stores centrally located within the institution’s premises, while MOUAU retail stores were largely situated along the campus periphery (Figure 3).

## 4. Discussion

The food environment plays a significant role in shaping individuals’ dietary habits and food choices. This study assessed the campus food environments in three different Nigerian universities. The dominance of food retail stores and advert count by UNN, followed by UI and MOUAU, is supported by findings from previous research, which stated that urbanisation and the size of an institution could lead to the availability of numerous food outlets in the university environment, including their corresponding food-related promotions [17,33]. While estimates indicate that the student populations of UNN, UI, and MOUAU are approximately 48,000, 35,000, and 30,000, respectively [34,35,36], UI is situated in an urban area, whereas UNN is located in a relatively more urbanised semi-urban area than MOUAU.

The preponderance of sugar-sweetened and alcoholic beverages, and the negligible proportions of fruit, vegetable, and dairy product categories, reflect the observed predominance of unhealthy food advertisements. This is not surprising, as branded alcoholic and non-alcoholic beverages are top-rated globally for their high advertising expenditure [37]. These advertising investments are often directed toward young people, with schools serving as strategic settings for targeted food marketing because of their high concentration of adolescents and young adults seeking convenient alternatives for food preparation and consumption [6,7,17,38]. The observed pattern of advertising aligns well with similar studies, which reported that posters/banners, food store premises, and promotional characters were common marketing approaches employed in this region [15,16]. The limited or unavailability of healthier, natural foods in these retail stores compares closely with multiple studies across different countries, including Spain, New Zealand, Australia, and the USA, which reported low availability of healthy foods on university campuses [39,40,41,42]. University food environments are typically characterised by cafeterias, canteens, and on-campus provision stores that predominantly sell branded foods, while healthier alternatives are often accessible only in off-campus markets located at some distance from institutional premises. Consistent with previous studies [17,26,43], this spatial and retail arrangement likely explains the low availability of whole foods such as fresh meat, fish, and poultry as well as fruits and vegetables with shorter shelf lives, while ready-to-eat healthy options like eggs and milk/dairy products were more commonly available.

The present study demonstrates that healthy foods are not only unavailable but also less affordable compared with their unhealthy alternatives. This finding is consistent with a plethora of evidence employing diverse evaluation metrics, which show that unhealthier products, particularly those high in fat and sugar, are cheaper than healthier options [26,43], with wider disparities observed in low-income settings [43,44].

Thus, these overall low to moderate availability and relatively affordability of whole, natural healthy foods compared to their branded unhealthy counterparts together with the widespread unhealthy food advertisement, highlight the need to strengthen the implementation and enforcement of existing food marketing regulations [45], including the ₦10 (US $0.02) per-litre tax on non-alcoholic beverages [46], and other government incentives aimed at improving access to healthy foods. Continued exposure to unhealthy food environments may reinforce students’ food cravings and influence their food choices and dietary habits. Although our study did not assess dietary intake and food choice behaviours among university students, previous studies have reported associations between unhealthy food environments and dietary behaviours and weight-related outcomes [18,19,20]. Overall, the findings through exposition of the gaps in campus food environment reflect the need for creating healthier campus food environments as part of broader strategies to promote healthy diets among university students in Nigeria.

## 5. Conclusions

This study revealed the predominance of unhealthy food advertisements clustered within food sales points, and a low degree of retail store healthiness, characterised by unavailable and relatively less affordable healthier foods among participating institutions. When disaggregated by institution, UNN had substantially more diverse adverts and available and affordable healthy foods than other institutions (*p* < 0.001).

There is a need for concerted efforts by the government and all stakeholders to strengthen food procurement practices, improve the availability and affordability of healthier foods, create structured retail pathways for informal vendors such as open-air produce sellers, and enhance the implementation and enforcement of food marketing regulations, specifically the restriction of unhealthy food advertisement within and around university campuses, which may help support healthier campus food environments. Future studies should be designed to assess more informal retail settings, explore the institutional and contextual factors underlying differences in campus food environments, and examine their associations with students’ dietary behaviours and health outcomes.

## 6. Limitations of the Study

Although this study provides a comprehensive assessment of the healthiness of campus food environments by evaluating both outdoor food advertising and the in-store availability and affordability of foods across three Nigerian universities, several limitations should be acknowledged.

First, although the selected universities provided variation in geographical location, rural–urban setting, and campus context, they were purposively selected rather than randomly sampled. Consequently, the findings should not be interpreted as representative of all Nigerian universities, and caution is warranted when generalising the results to institutions in other regions or with different contextual characteristics.

Given that the study examined differences across institutions across a range of food environment indicators. The large number of statistical tests may increase the likelihood of Type I error. Accordingly, statistically significant findings should be interpreted alongside the overall pattern and consistency of the results rather than as isolated comparisons.

Also, the University of Ibadan was not included in the outdoor food advertisement audit because of logistical and operational constraints. This may have limited comparisons of food advertising environments across all three institutions.

The unique characteristics of the Nigerian food system required minor adaptations to the INFORMAS and NEMS-S-MED classification frameworks. Some locally available foods that did not align with predefined food categories were either assigned to the closest comparable category or excluded where appropriate, which may have affected the classification of a small number of food items.

Finally, open-air food vendors and specialised retail outlets were not included because they typically stocked a limited range of food groups. As these outlets may provide healthier food options, including fresh fruits, vegetables, meat, and fish, their exclusion may have resulted in an incomplete assessment of the broader campus food environment.

## Figures and Tables

**Table 1:** Advertisement type and settings.

	MOUAU (n = 16) F (%)	UNN (n = 60) F (%)	Total (n = 76) F (%)	Chi-Square	*p*-Value
**Size of advert**					
Small	16 (100)	35 (58.3)	51 (67.1)	9.935	0.007
Medium	-	22 (36.7)	22 (28.9)		
Large	-	3 (5.0)	3 (3.9)		
**Type of advert**					
Billboard	1 (6.3)	1 (1.7)	2 (2.6)	10.369	0.035
Posters/banners	15 (93.8)	34 (56.7)	49 (64.5)		
Painted building/wall	-	5 (8.3)	5 (6.6)		
Free-standing	-	18 (30.0)	18 (23.7)		
Others (opener and fridge)	-	2 (3.3)	2 (2.6)		
**Settings of the ad**					
Food shop	13 (81.3)	46 (76.7)	59 (77.6)	5.99	0.11
Food road	3 (18.8)	3 (5.0)	6 (7.9)		
Building	-	8 (13.3)	8 (10.5)		
Others	-	3 (5.0)	3 (3.9)		
**Presence of promotional character**					
Yes	-	30 (50.0)	30 (39.5)	13.217	<0.001
No	16 (100)	30 (50.0)	46 (60.5)		
**Forms of promotional character**					
Cartoon/company-owned character	-	13 (21.7)	13 (17.1)	13.217	0.040
Licensed character	-	13 (21.7)	13 (17.1)		
Amateur sportsperson	-	1 (1.7)	1 (1.3)		
Celebrity (non-sports)	-	1 (1.7)	1 (1.3)		
Non-sports/historical events	-	1 (1.7)	1 (1.3)		
For kids		1 (1.7)	1 (1.3)		
Not applicable	16 (100)	30 (50.0)	46 (60.5)		
**Premium Offers**					
Contests	-	2 (3.3)	2 (2.6)	12.505	0.130
Extra or other	-	4 (6.7)	4 (5.3)		
Extra or another limited edition	-	1 (1.7)	1 (1.3)		
Gift or collectable		4 (6.7)	4 (5.3)		
Limited edition	-	13 (21.7)	13 (17.1)		
Pay 2 take 3 or other	-	1 (1.7)	1 (1.3)		
Price discount	-	3 (5.0)	3 (3.9)		
Social charity	-	1 (1.7)	1 (1.3)		
Not applicable	16 (100)	31 (51.7)	47 (61.8)		

**Table 2:** Healthiness of food advertised by school.

	MOUAU F (%)	UNN F (%)	Total F (%)	Chi-Square	*p*-Value
**Number of foods advertised**					
Mean (SD)	1.44 (1.365)	1.68 (1.513)	1.63 (1.477)	F = 0.347	ANOVA *p* = 0.558
Min.	1	1	1		
Max.	6	8	8		
Count	24	96	120		
**Healthiness of products**					
Healthy	3 (12.5)	6 (6.3)	9 (7.5)	3.010	0.222
Non-healthy	21 (87.5)	82 (85.4)	103 (85.8)		
Miscellaneous	0 (0.0)	8 (8.3)	8 (6.7)		
**Total**	24 (100)	96 (100)	120 (100)		

**Table 3:** Availability of healthy foods at the campus retail stores.

	MOUAU (n = 45) F (%)	UNN (n = 54) F (%)	UI (n = 46) F (%)	Total (n = 145) F (%)	H (df = 2)	*p*-Value
**Variety of fresh fruits**						
None	11 (24.4)	-	14 (30.4)	25 (17.2)	63.529	<0.001
1–5 varieties	32 (71.1)	19 (35.2)	31 (67.4)	82 (56.6)		
6–10 varieties	1 (2.2)	26 (48.1)	1 (2.2)	28 (19.3)		
>10 varieties	1 (2.2)	9 (16.7)	-	10 (6.9)		
**Variety of fresh vegetable**						
None	10 (22.2)	1 (1.9)	16 (34.8)	27 (18.6)	43.522	<0.001
1–5 varieties	30 (66.7)	25 (46.3)	28 (60.9)	83 (57.2)		
6–10 varieties	5 (11.1)	19 (35.2)	2 (4.3)	26 (17.9)		
>10 varieties	-	9 (16.7)	0 (0.0)	9 (6.2)		
**Variety of frozen vegetables**						
None	10 (22.2)	1 (1.9)	16 (34.8)	27 (18.6)		
1–5 varieties	35 (77.8)	44 (81.5)	30 (65.2)	109 (75.2)	28.149	<0.001
6–10 varieties	-	9 (16.7)	0 (0.0)	9 (6.2)		
>10 varieties	-					
**Variety of fresh fish**						
None	10 (22.2)	1 (1.9)	15 (32.6)	26 (17.9)		
1–5 varieties	33 (73.3)	46 (85.2)	30 (65.2)	109 (75.2)	19.510	<0.001
6–10 varieties	2 (4.4)	7 (13.0)	1 (2.2)	10 (6.9)		
>10 varieties	-	-	-	-		
**Variety of frozen fish**						
None	11 (24.4)	2 (3.7)	15 (32.6)	28 (19.3)		
1–5 varieties	29 (64.4)	48 (88.9)	31 (67.4)	108 (74.5)	14.010	<0.001
6–10 varieties	5 (11.1)	4 (7.4)	-	9 (6.2)		
>10 varieties	-	-	-	-		
**Raw nut**						
Available	11 (24.4)	53 (98.1)	-	64 (44.1)	106.572	<0.001
Not Available	34 (75.6)	1 (1.9)	46 (100)	81 (55.9)		
**100% juice**						
Available	-	1 (1.9)	10 (21.7)	11 (7.6)	19.236	<0.001
Not Available	45 (100)	53 (98.1)	36 (78.3)	134 (92.4)		
**100% whole-grain bread**						
Available	5 (11.1)	2 (3.7)	-	7 (4.8)	6.305	0.043
Not Available	40 (88.9)	52 (96.3)	46 (100)	138 (95.2)		
**Low-sugar cereals**						
Available	23 (51.1)	14 (25.9)	4 (8.7)	41 (28.3)	20.272	<0.001
Not Available	22 (48.9)	40 (74.1)	42 (91.3)	104 (71.7)		
**Any type of milk**						
Available	31 (68.9)	48 (88.9)	33 (71.7)	112 (77.2)	6.699	0.035
Not Available	14 (31.1)	6 (11.1)	13 (28.3)	33 (22.8)		
**Skimmed yoghurt**						
Available	12 (26.7)	3 (5.6)	1 (2.2)	16 (11.0)	16.418	<0.001
Not Available	33 (73.3)	51 (94.4)	45 (97.8)	129 (89.0)		
**Cheese**						
Available	-	25 (46.3)	-	25 (17.2)	50.556	<0.001
Not Available	45 (100)	29 (53.7)	46 (100)	120 (82.8)		
**Eggs**						
Available	37 (82.2)	48 (88.9)	32 (69.6)	117 (80.7)	6.009	0.050
Not Available	8 (17.8)	6 (11.1)	14 (30.4)	28 (19.3)		
**Extra-virgin oil**						
Available	16 (35.6)	47 (87.0)	2 (4.3)	65 (44.4)	70.451	<0.001
Not Available	29 (64.4)	7 (13.0)	44 (95.7)	80 (55.2)		
**Whole rice**						
Available	9 (20.0)	37 (68.5)	2 (4.3)	48 (33.1)	50.896	<0.001
Not Available	36 (80.0)	17 (31.5)	44 (95.7)	97 (66.9)		
**White rice**						
Available	14 (31.1)	37 (68.5)	17 (37.0)	68 (46.9)	16.465	<0.001
Not Available	31 (68.9)	17 (31.5)	29 (63.0)	77 (53.1)		
**Legumes**						
Available	13 (28.9)	25 (46.3)	17 (37.0)	55 (37.9)	3.164	0.206
Not Available	32 (71.1)	29 (53.7)	29 (63.0)	90 (62.1)		
**Red meat**						
Available	7 (15.6)	30 (55.6)	4 (8.7)	41 (28.3)	31.880	<0.001
Not Available	38 (84.4)	24 (44.4)	42 (91.3)	104 (71.7)		
**Poultry**						
Available	6 (13.3)	33 (61.1)	3 (6.5)	42 (29.0)	43.424	<0.001
Not Available	39 (86.7)	21 (38.9)	43 (93.5)	103 (71.0)		
**Canned tuna**						
Available	4 (8.9)	10 (18.5)	19 (41.3)	33 (22.8)	14.377	<0.001
Not Available	41 (91.1)	44 (81.5)	27 (58.7)	112 (77.2)		

**Table 4:** Affordability of healthy foods in retail stores.

	MOUAU (n = 45) F (%)	UNN (n = 54) F (%)	UI (n = 46) F (%)	Total (n = 145) F (%)	H (df = 2)	*p*-Value
**Non-alcoholic beverages**						
Diet soda ≤ regular	3 (6.7)	25 (46.3)	20 (43.5)	48 (33.1)	20.539	<0.001
100% juice ≤ juice drinks	-	-	-	-		
Regular/juice drink is cheaper	42 (93.3)	29 (53.7)	12 (26.1)	83 (57.2)		
Not applicable	-	-	14 (30.4)	14 (9.7)		
**Bread, cereals, and baked goods**						
Low-sugar cereals ≤ regular	-	24 (44.4)	2 (4.3)	26 (17.9)	41.109	<0.001
Regular is cheaper	45 (100)	30 (55.6)	27 (58.7)	102 (70.3)		
Not applicable	-	-	17 (37.0)	17 (11.7)		
**Milk and dairy products**						
Skimmed- or low-fat milk < whole milk	-	17 (31.5)	19 (41.3)	36 (24.8)	23.560	<0.001
Skimmed- or low-fat milk = whole milk	-	2 (3.7)	-	2 (1.4)		
Whole milk is cheaper	45 (100)	35 (64.8)	13 (28.3)	93 (64.1)		
Not applicable	-	-	14 (30.4)	14 (9.7)		
**Rice**						
Whole rice ≤ white rice	19 (42.2)	19 (35.2)	-	38 (26.2)	24.386	<0.001
White rice is cheaper	26 (57.8)	35 (64.8)	12 (26.1)	73 (50.3)		
Not applicable	-	-	34 (73.9)	34 (23.4)		
**Meat and meat products**						
Poultry meat ≤ red meat	4 (8.9)	16 (29.6)	1 (2.2)	21 (14.5)	16.652	<0.001
Red meat is cheaper	41 (91.1)	38 (70.4)	3 (6.5)	82 (56.6)		
Not applicable	-	-	42 (91.3)	42 (29.0)		

**Table 5:** Summary of healthy food availability, affordability, and overall healthiness of in-store food environments across university campuses.

	MOUAU (n = 45) F (%)	UNN (n = 54) F (%)	UI (n = 46) F (%)	Total (n = 145) F (%)	H (df = 2)	*p*-Value
**Degree of availability**					55.253	<0.001
Low availability (0–7)	7 (15.6)	-	14 (30.4)	21 (14.5)		
Moderately low availability (8–14)	18 (40.0)	11 (20.4)	25 (54.3)	54 (37.2)		
Medium availability (15–22)	19 (42.2)	20 (37.0)	7 (15.2)	46 (31.7)		
High availability (23–37)	1 (2.2)	23 (42.6)	-	24 (16.6)		
**Degree of Affordability (Price)**					28.105	<0.001
Low affordability (0–3)	43 (95.6)	27 (50.0)	36 (78.3)	106 (73.1)		
Moderate affordability (4–7)	2 (4.4)	20 (37.0)	10 (21.7)	32 (22.1)		
High affordability (8–12)	-	7 (13.0)	-	7 (4.8)		
**Degree of Healthiness**					50.656	<0.001
Low (0–16)	30 (66.7)	8 (14.8)	37 (80.4)	75 (51.7)		
Moderate (17–33)	15 (33.3)	41 (75.9)	9 (19.6)	65 (44.8)		
High (34–49)	-	5 (9.3)	-	5 (3.4)		

## Data Availability

The code and documentation for the spatial analysis are publicly available at https://github.com/Gidiblings/campus_spatial_food_environment_Nig (accessed on 8 August 2026).

## Abbreviations

UNN	University of Nigeria, Nsukka
MOUAU	Michael Okpara University of Agriculture, Umudike,
UI	University of Ibadan
INFORMAS	International Network for Food and Obesity/Non-Communicable Diseases Research, Monitoring and Action Support
NEMS-S-MED	Nutrition Environment Measures Survey in Stores for Mediterranean Contexts
GPS	Global Positioning System
